# Interstitial pneumonia induced by sorafenib in a patient with hepatocellular carcinoma: An autopsy case report

**DOI:** 10.3892/ol.2015.2934

**Published:** 2015-02-05

**Authors:** TAKASHI YAMAGUCHI, TOSHIHITO SEKI, CHIKA MIYASAKA, RYOSUKE INOKUCHI, RINAKO KAWAMURA, YUUTAKU SAKAGUCHI, MIKI MURATA, KOICHI MATSUZAKI, YORIKA NAKANO, YOSHIKO UEMURA, KAZUICHI OKAZAKI

**Affiliations:** 1 Department of Gastroenterology and Hepatology, Kansai Medical University, Osaka 570-8507, Japan; 2 Liver Disease Center, Kansai Medical University Takii Hospital, Osaka 570-8507, Japan; 3 Department of Diagnostic Pathology, Kansai Medical University, Osaka 570-8507, Japan

**Keywords:** diffuse alveolar damage, hepatocellular carcinoma, drug-induced interstitial pneumonia, molecular-targeted agent, sorafenib

## Abstract

Sorafenib is a multikinase inhibitor currently approved in Japan for the treatment of unresectable hepatocellular carcinoma. Interstitial pneumonia induced by sorafenib may have a fatal outcome, and therefore, has recently been the focus of many studies. The current report presents an autopsy case of diffuse alveolar damage (DAD) that occurred in a 59-year-old male, who had been treated with sorafenib. The patient had been given sorafenib for six months and had exhibited no respiratory symptoms during this time. However, 19 days after sorafenib treatment was resumed, acute interstitial pneumonia developed. In previously reported cases, the first symptoms of pulmonary toxicity appeared following a limited treatment duration with sorafenib; this was in contrast to the patient in the current study, who developed the first symptoms after eight months. We therefore conclude that physicians must be aware of interstitial pneumonia as a potential pulmonary toxicity associated with sorafenib treatment when treatment with sorafenib is resumed, even after prolonged use. In addition, to best of our knowledge, this is the first case of a postmortem examination reported in patient with interstitial pneumonia induced by sorafenib treatment.

## Introduction

Sorafenib (Bayer Pharmaceuticals, West Haven, CT, USA) is a multikinase inhibitor, which functions by blocking tumor-cell proliferation and angiogenesis (1). In cases of advanced hepatocellular carcinoma (HCC) where patients received sorafenib treatment, almost a 3-month median survival benefit was reported, as compared with patients receiving a placebo (2).

Common adverse side effects of sorafenib treatment include diarrhea, weight loss, skin rash (including hand-foot skin reactions), fatigue, and hypertension. Additionally, a number of cases of sorafenib-induced interstitial pneumonia have also been reported (3–5). Safety information for sorafenib therapy in patients with HCC was presented in Japan in October 2012, and six cases of acute respiratory failure were reported among 1,045 patients with HCC who had been treated with sorafenib (6). The current study describes an autopsy case of interstitial pneumonia that developed after the long-term treatment of a patient with advanced HCC with sorafenib. Written informed consent was obtained from the family of the patient.

## Case report

**Figure 1:**
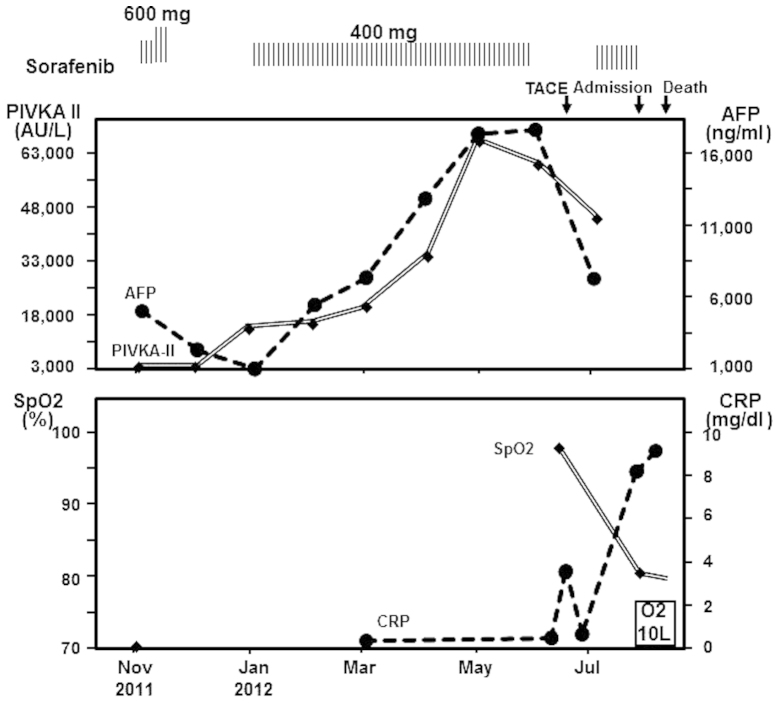
Clinical course of the patient after the administration of sorafenib. Serum AFP and PIVKA-II reduced after TACE. On day 19 of sorafenib readministration, the patient developed progressive dyspnea, and was admitted to hospital. The oxygen saturation was 81% despite oxygen supplementation. There was a clinical worsening, and the patient succumbed to the disease three days after admission. AFP, α-fetoprotein; PIVKA-II, protein induced by vitamin K absence or antagonist II; TACE, transcatheter arterial chemoembolization; CRP, C-reactive protein.

**Figure 2:**
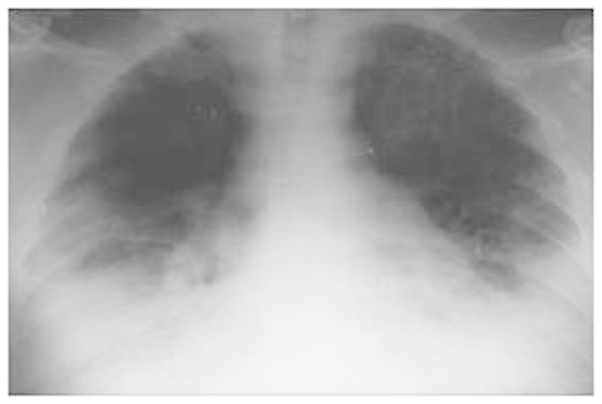
Chest X-ray on admission.

A 59-year-old male with hepatitis C virus-related, multinodular HCC, exhibited progressive disease following eight sessions of transarterial chemoembolization (TACE) and four sessions of ablation therapy over the previous 15 years Kansai Medical University Takii Hospital (Osaka, Japan) and was admitted to the Department of Gastroenterology and Hepatology, Kansai Medical University (Osaka, Japan). Radiological studies showed growth of the tumor in the right lobe of the liver with several intrahepatic metastases, and further metastases to the lung. Although the patient had smoked until 25 years of age, no respiratory symptoms prior to the administration of sorafenib were observed. Additional medication at the time of commencing sorafenib treatment included ursodeoxycholic acid, branched-chain amino acid-containing pharmaceutical granular preparation, and tamsulosin hydrochloride. Fig. 1 shows the clinical course following the administration of sorafenib. Due to the patient’s general state of health and Child-Pugh class B (score 7), palliative treatment with sorafenib (400 mg daily) was initiated in November 2011. After one week, the dosage was increased to 600 mg/day. Two weeks following initiation, the administration of sorafenib was discontinued due to hand-foot-skin reaction, and was resumed at a dose of 400 mg/day four weeks later. After five months, sorafenib treatment was discontinued again due to the patient being treated with TACE, and subsequently resumed at 400 mg/day after four weeks. At 19 days following the treatment resumption, the patient developed progressive dyspnea and fever, with worsening general weakness, and presented to the emergency department of Kansai Medical University Takii Hospital with dyspnea, cough and fever. Analysis of the vital signs showed a normal blood pressure of 124/65 mmHg (normal range, 100–129/60–80 mmHg), respiratory rate of 20 breaths/min (normal range, 12–15 breaths/min), pulse of 120 beats/min (normal range, 60–85 beats/min), and body temperature of 37.5°C (normal range, 35.0–37.0°C). Respiratory crackles were audible in the bilateral lower lung fields; the patient was anemic and icteric. The air pulse oximetric saturation was 81% (normal limit, >92%); arterial blood gas analysis showed a PaO_2_ of 62.5 mmHg (normal range, 80–100 mmHg); PaCO_2_ of 28.5 mmHg (normal range, 35–45mmHg) and pH 7.39 (normal range, 7.35–7.45), despite oxygen supplementation. Laboratory studies showed marked leukocytosis with a white blood cell count of 7,300 cells/μl (normal range, 3,500–8,500 cells/μl), a neutrophil level of 6,607 cells/μl (normal range, 1,470–6,545 cells/μl) and an elevated C-reactive protein level of 8.05 mg/dl (normal limit, <0.3 mg/dl); elevated aspartate transaminase concentration of 237 IU/l (normal range, 13–35 IU/l), and alanine transaminase concentration of 89 IU/l (normal range, 5–35 IU/l) (Table I). Chest X-ray radiography revealed heart enlargement and bilateral pleural effusion, leading to a diagnosis of acute heart failure (Fig. 2). Sorafenib treatment was discontinued on admission to Kansai Medical University Takii Hospital as oral administration was difficult. The patient developed rapidly worsening dyspnea and hypoxia in spite of therapy with diuretic treatment and providing oxygen, and the patient succumbed to the disease three days following admission. The patient had declined mechanical ventilation. The autopsy was conducted with the consent of the family.

### Autopsy findings

**Figure 3:**
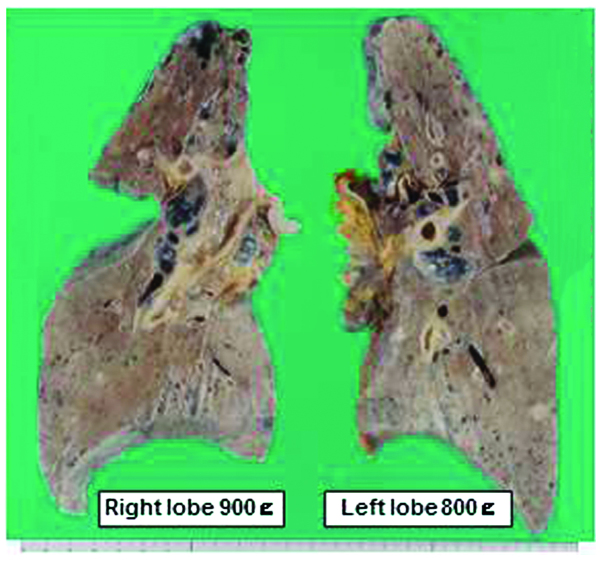
Gross features of the lungs at autopsy.

**Figure 4:**
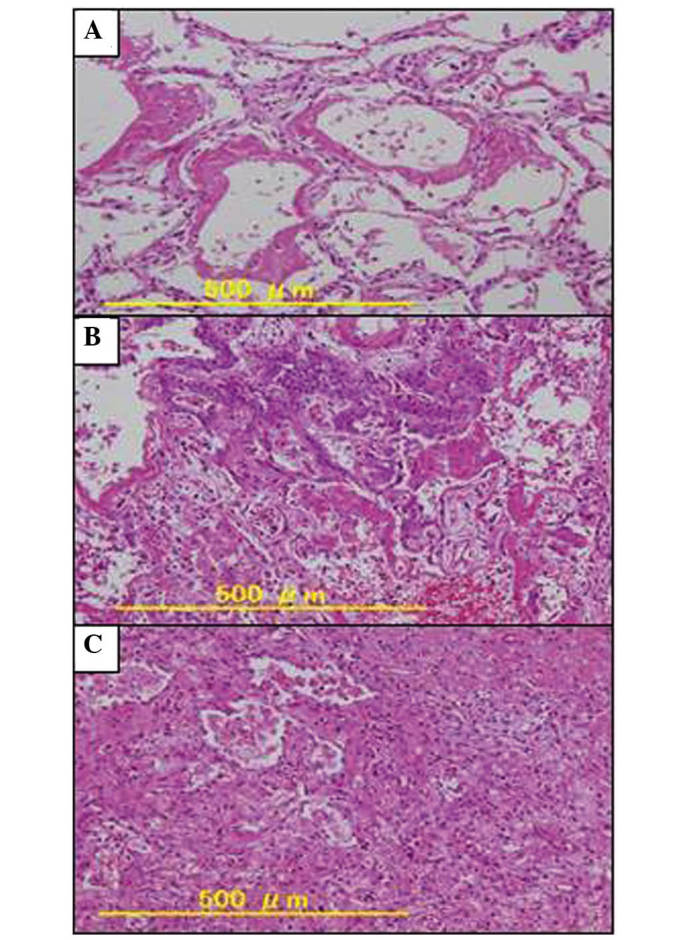
Microscopic findings in the lungs. Diffuse alveolar damage with hyaline membranes superimposed on a fibrotic lung background (hematoxylin and eosin stain). (A) Early exudative stage: Injury of type II pneumocytes with sloughing into alveolar lumens and hyaline membrane formation. (B) Proliferative stage: Organization of exudates composed of proliferating type II pneumocytes and fibroblasts with squamous metaplasia. (C) Late organizing fibrotic phase: Interstitial fibrosis with widening of alveolar septa and disappearance of the hyaline membranes.

Gross findings of the autopsied liver revealed cirrhosis, and multiple nodular lesions, with the largest measuring 4 cm in diameter, were homogeneously yellow-white to green. Histologically, the lesions were composed of HCC and intrahepatic cholangiocarcinoma (ICC) elements. A histological diagnosis of intermixed HCC-ICC was determined. The HCC element revealed a proliferating trabecular pattern with bile production, corresponding to moderate differentiation as Edmondson’s grade II (7). The ICC element was well-differentiated, forming a well-developed gland. The largest mass was widely necrotic and exhibited fibrotic changes, considered to be the effect of sorafenib treatment and TACE; the remaining masses were ICC elements. Metastasis to the lungs, hilar lymph nodes, and mediastinal lymph nodes was observed. Furthermore, necrosis was identified, partially due to sorafenib treatment, and the residual regions showed ICC elements only. On autopsy, the lungs were swollen, with a combined weight of 1,700 g, and consolidated with a diffusely glistening spongy cut surface (Fig. 3). Histologically, the alveoli were obliterated by the hyaline membranes and organization of exudates with proliferation of fibroblasts, indicating diffuse alveolar damage (DAD). Notably, the bilateral lungs showed a diffusely different phases of DAD, with the hyaline membrane producing an early exudative phase (Fig. 4A), a proliferative phase (Fig. 4B), and late organizing fibrotic phase (Fig. 4C). The mixed features of various phases were proposed to correspond to drug-induced DAD. Honeycombing was not observed. The findings did not indicate other organisms such as bacteria, cytomegalovirus, *Pneumocystis jirovecii*, and fungus. In addition, no vascular changes of pulmonary hypertension with a plexiform lesion were identified, however, a number of arterializations of small blood vessels were revealed. No evidence of recent myocardial infarction or acute cardiac decompensation was identified. Due to all of the results, a clinical diagnosis of fatal interstitial pneumonia associated with sorafenib treatment was determined.

## Discussion

The current study presents an autopsy case involving a patient with advanced HCC who developed rapidly progressive interstitial lung disease following resumption of treatment with sorafenib. In the absence of other etiologies, and due to the autopsy findings, this patient was considered to have sorafenib-induced interstitial pneumonia.

Histologically, the autopsied lungs revealed DAD, which is the morphological precursor to acute interstitial pneumonia and is characterized by a rapid and fatal clinical course. DAD manifests clinically as acute respiratory distress syndrome (ARDS) (8); it may be observed in sepsis, shock, trauma, severe ARDS, and idiopathic cases with undetected etiological factors as well as acute exacerbations of chronic interstitial lung diseases. While diffuse bilateral opacity is often observed on lung radiology, numerous cases display deep hypoxemia that requires mechanical ventilation; the mortality rate is 43–50% (9,10). A number of drugs have been associated with lung injury with a DAD pattern. The clinical features of lung toxicity are not specific (dyspnea, cough, fever, pulmonary infiltrates), and the differential diagnosis includes infection, relapse of the underlying disease, pulmonary edema, and changes due to oxygen or radiation (11). The pathological findings of drug-related DAD are also nonspecific and the diagnosis is one of exclusion (12). In the present case, the clinical history and exclusion of other causative factors indicate that sorafenib is the cause of lung injury.

Treatment with a number of molecular-targeted agents, including gefitinib, erlotinib, imatinib, and bortezomib, has been associated with pulmonary toxicity (13). However, the underlying mechanisms of how these molecular-targeted agents induce interstitial pneumonia remain unknown. The reduction of intrapulmonary vascular endothelial growth factor (VEGF) levels in the early stages of lung injury and normalization following recovery in ARDS have been confirmed in numerous studies (14,15), as VEGF acts as a growth and anti-apoptotic factor on alveolar epithelial cells, in addition to its known effects on endothelial cells (16). Therefore, the pulmonary toxicity induced by sorafenib treatment may be associated with its mechanism of antitumor activity, involving the inhibition of the VEGF signaling pathway.

In previously reported cases, the initial symptoms of pulmonary toxicity appeared after a limited treatment duration with sorafenib (one to six weeks) (3–5). By contrast, the present case showed delayed onset after eight months of sorafenib treatment. The patient had been treated with sorafenib for six months with no respiratory symptoms prior to resuming sorafenib treatment. After 19 days of resuming the treatment, however, the patient developed acute interstitial pneumonia. A number of immune-related mechanisms in the interstitial pneumonia may be induced by sorafenib.

In conclusion, severe respiratory failure with a histological pattern of DAD may develop following resumption of treatment with sorafenib. Therefore, physicians must be aware of interstitial pneumonia as a potential pulmonary toxicity associated with sorafenib treatment, when a patient resumes treatment with sorafenib, even after prolonged use.

## Figures and Tables

**Table I:** Laboratory data on admission.

Marker	Measurement	Range
Hematology		
WBC	7,300/μl	
Neutro	90.5%	
Lympho	4.5%	
Mono	4.0%	
Eosino	0.5%	
Baso	0.5%	
RBC	350×10^4^/μl	↓
Hb	8.6g/dl	↓
Ht	28.1%	↓
Plt	8.9×10^4^/μl	↓
Coagulation		
PT	34%	↓
INR	1.79	
Biochemistry		
AST	237 U/l	↑
ALT	89 U/l	↑
T-Bil	2.6 mg/dl	↑
D-Bil	1.5 mg/dl	↑
ALP	428 U/l	↑
γ-GTP	17 U/l	
LDH	1,119 U/l	↑
TP	6.6 g/dl	
Alb	2.1 g/dl	↓
BUN	25 mg/dl	↑
Creatine	0.92 mg/dl	
CRP	8.050 mg/dl	↑
NH_3_	48 μg/ml	
Tumor markers		
AFP	6,139.0 ng/ml	↑
AFP-L3	65.1%	↑
PIVKA-II	27,800 AU/l	↑
Blood gas analysis		
pH	7.394	
pCO_2_	28.5 mgHg	↓
pO_2_	62.5 mgHg	↓
HCO_3_^−^	17.0 mEq/l	↓

WBC, white blood cells; Hb, hemoglobin; Ht, hematocrit; Plt, platelet; PT, prothrombin time; INR, international normalized ratio; AST, aspartate transaminase; ALT, alanine transaminase; T-Bil, total bilirubin; D-Bil, direct bilirubin; ALP, alkaline phosphatase; γ-GTP, γ-glutamyl transferase; LDH, lactate dehydrogenase; TP, total protein; Alb, albumin; BUN, blood urea nitrogen; CRP, C-reactive protein; AFP, α-fetoprotein; AFP-L3, AFP-L3 isoform; PIVKA-II, proteins induced by vitamin K absence or agonist-II; ↓, lower than normal range; ↑, higher than normal range.
